# Simultaneous viscosity and density measurement of small volumes of liquids using a vibrating microcantilever†
†Electronic supplementary information (ESI) available. See DOI: 10.1039/c6an02674e



**DOI:** 10.1039/c6an02674e

**Published:** 2017-03-20

**Authors:** A. F. Payam, W. Trewby, K. Voïtchovsky

**Affiliations:** a Department of Physics , Durham University , Durham , UK . Email: kislon.voitchovsky@durham.ac.uk

## Abstract

Many industrial and technological applications require precise determination of the viscosity and density of liquids. Such measurements can be time consuming and often require sampling substantial amounts of the liquid. These problems can partly be overcome with the use of microcantilevers but most existing methods depend on the specific geometry and properties of the cantilever, which renders simple, accurate measurement difficult. Here we present a new approach able to simultaneously quantify both the density and the viscosity of microliters of liquids. The method, based solely on the measurement of two characteristic frequencies of an immersed microcantilever, is completely independent of the choice of a cantilever. We derive analytical expressions for the liquid's density and viscosity and validate our approach with several simple liquids and different cantilevers. Application of our model to non-Newtonian fluids shows that the calculated viscosities are remarkably robust when compared to measurements obtained from a standard rheometer. However, the results become increasingly dependent on the cantilever geometry as the frequency-dependent nature of the liquid's viscosity becomes more significant.

## Abstract 2

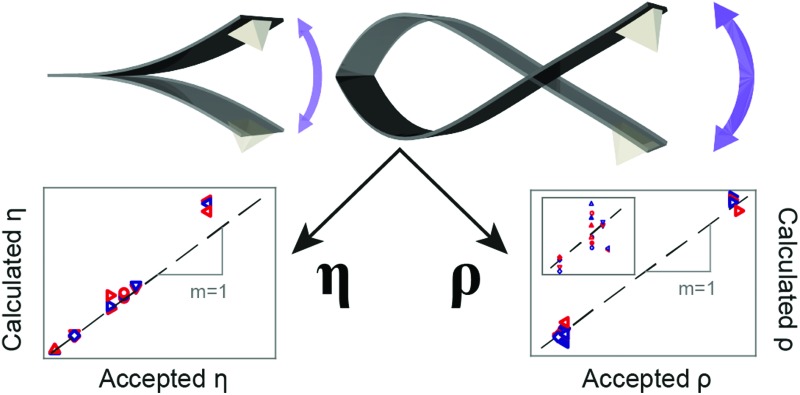
An analytical model is derived to calculate the viscosity and density of small volumes of fluid from the resonance frequencies of an immersed microcantilever. Its accuracy is verified on simple and non-Newtonian fluids.

## Introduction

Accurate and rapid determination of the density and viscosity of liquids is central to countless industrial, technological and scientific processes. Applications range from oil and lubricant characterization in the petroleum industry^1^ to chemical engineering,^2^ quality control in food science^3^ and biomedical research, in particular for the detection and diagnosis of diseases from bodily fluids.^4–6^ One of the challenges faced by conventional measurement methods is the need for large volumes of liquid. Standard rheometers can provide accurate viscosity measurements over an extensive range of temperatures and pressures^7^ but they require relatively large samples, typically several millilitres or more. Measurement methods based on acoustic waves,^8^ tuning forks^9^ or microfluidics^10^ have made it possible to probe smaller liquid volumes, but the liquid's density and viscosity cannot be measured simultaneously; one quantity is needed in order to deduce the other from the experimental data. To overcome this limitation, sensors based on microcantilevers have been proposed.^11–21^ These sensors typically require only small volumes (tens of microlitres) of fluid^22^ and are able to determine the viscosity and density simultaneously,^11,12,15,18–20^ making them particularly attractive for lab-on-chip-type diagnostic devices. Measurements effectively quantify changes in the dynamic response of the microcantilever upon immersion into the liquid examined. Fitting the experimental results with theoretical models yields the rheological parameters of the liquid, but the accuracy of the results depends crucially on the quality of the theoretical model, and the ability to implement it fast and robustly. Developing a suitable model is hence far from trivial, because it requires taking into account the coupling between a vibrating cantilever of a given geometry and the surrounding liquid. This is usually characterized by the so-called hydrodynamic function of the cantilever, which in turn depends on the rheological properties of the liquid.

Early developments used significant simplifications such as a spherical model for cantilevers^23^ or an inviscid fluid^24–26^ resulting in large errors or limited applicability. Part of the difficulty comes from the need to take into account the exact geometry of the cantilever and its mechanical properties to precisely determine its hydrodynamic function. By measuring a cantilever's thermal spectrum (that is, its frequency response to the thermal excitation in the liquid) and fitting it to a simple harmonic oscillator model,^27,28^ it is in principle possible to determine the unknown geometrical factors,^29^ but this method fails in highly viscous environments. The first semi-analytical model explicitly taking into account the geometry and properties of the cantilever to describe its behaviour in liquid was developed by Sader.^30^ The so-called Sader model provides acceptable agreement with experimental measurements,^31^ but at the cost of computationally intensive calculations and a detailed knowledge of the cantilever. Extending the Sader approach to higher resonances of the microcantilever^32,33^ makes it possible to overcome these difficulties and derive analytical expressions for the cantilever's hydrodynamic function solely based on the frequency of the different resonances.^34^ However, to experimentally reconstruct the hydrodynamic function of a fluid, at least the first three resonance frequencies of the immersed cantilever are needed, something often challenging to measure experimentally in highly viscous liquids.^35^ Once the hydrodynamic function is known, it can be inverted to derive the viscosity and density of the liquid. This step requires some approximations and different methods have been proposed^15–20^ with a typical reported accuracy of 20% when both rheological parameters are determined simultaneously.^15^

In this paper, we derive novel analytical expressions for the hydrodynamic function of the immersed cantilever. This allows us to propose analytical expressions for the viscosity and the density of the surrounding liquid using only two resonance frequencies of the microcantilever. Significantly, the expressions are fully independent of the type or geometry of the cantilever used, greatly simplifying the derivation of liquid's properties. Our derivation, based on the Euler–Bernoulli beam theory for an immersed cantilever, extends the expression of the hydrodynamic function while considering water as a reference. We test experimentally our analytical expressions over a range of liquids and with several different cantilevers, demonstrating differences smaller than 10% between predictions and experimental results for both density and viscosity. We also investigate the limitations of these models by probing idealised non-Newtonian mixtures of water and poly(ethylene) oxide (PEO). PEO is an uncross-linked polymer and as such represents a model bodily fluid that displays viscoelastic properties at high molecular weight due to the overlapping of neighbouring polymer coils.^36–40^

For the purpose of this study, we used an atomic force microscope (AFM) to conduct our measurements. Our results are general and can be implemented in any type of microcantilever-based device. We also expect our findings to contribute towards significant improvements in the booming field of AFM in liquid, in particular for the analysis of surface-coupled effects on the cantilever vibrations and for the investigation of liquid flow near liquid–solid interfaces.^41–58^

## Materials and methods

In order to verify the accuracy of the proposed expressions and analyse the effect of cantilever parameters and liquid properties on the fluid dissipation mechanisms, thermal spectra were recorded in six well-characterised liquids with different viscosities and densities, namely isopropanol, acetone, butanol, decane, bromoform and hexanol. All the liquids were purchased from Sigma-Aldrich (Dorset, UK) with a purity > 99% and used without further purification. The reference liquid was ultrapure water (18.2 MΩ, Merck-Milipore, Dorset, UK). Our model non-Newtonian fluid consisted of varying concentrations of 300 000 g mol^–1^ poly(ethylene) oxide (PEO) (Sigma-Aldrich, Dorset, UK) in water. The PEO was immersed in ultrapure water to a concentration of 3 wt% and dissolved using a magnetic stirrer at 700 RPM for 24 hours until a uniform milky solution was obtained. The solution was then decanted into several 2 mL Eppendorf tubes and centrifuged at 2500 RPM for 10 minutes to separate the PEO solution from the insoluble butylated hydroxytoluene (BHT) that the initial powder contained as an inhibitor. The then-clear fluid was removed with a pipette and bath-sonicated for 10 minutes to remove any dissolved air. The PEO solution was then diluted to the required concentration with ultrapure water and the resulting mixture was bath-sonicated for 10 minutes to ensure uniformity.

The measurements on pure liquids were conducted on an MFP-3D Infinity AFM, and the PEO mixtures were measured on a Cypher ES AFM with a temperature-controlled sample stage (both AFMs from Asylum Research, Santa Barbara, CA). For each liquid, we took thermal spectra with four different cantilevers (OMCL-RC800PSA, Olympus, Japan). The cantilevers are made of silicon-nitride and have different lengths and widths. The nominal geometrical and physical characteristics of the different cantilevers (hereafter referred to as C1–C4) are summarized in Table 1. For each measurement, the cantilever was naturally excited by the Brownian motion of the fluid surrounding it, and its vertical motion was detected using the AFM laser.

**Table 1:** Summary of the physical characteristics of different cantilevers (C1–C4) used for this study. The cantilevers have a 3 μm-high tip mounted at one extremity

Cantilever reference	Width (μm)	Length (μm)	Thickness (μm)	Spring const. (N m^–1^)
C1	40	100	0.8	0.76
C2	20	100	0.8	0.39
C3	40	200	0.8	0.10
C4	20	200	0.8	0.05

## Results and discussion

### Theory

The derivation of the analytical expressions for viscosity and density is presented hereafter. For the sake of clarity, only the main steps and the results are presented. The detailed step-by-step derivation of the different results is given in the ESI.†

**Fig. 1:**
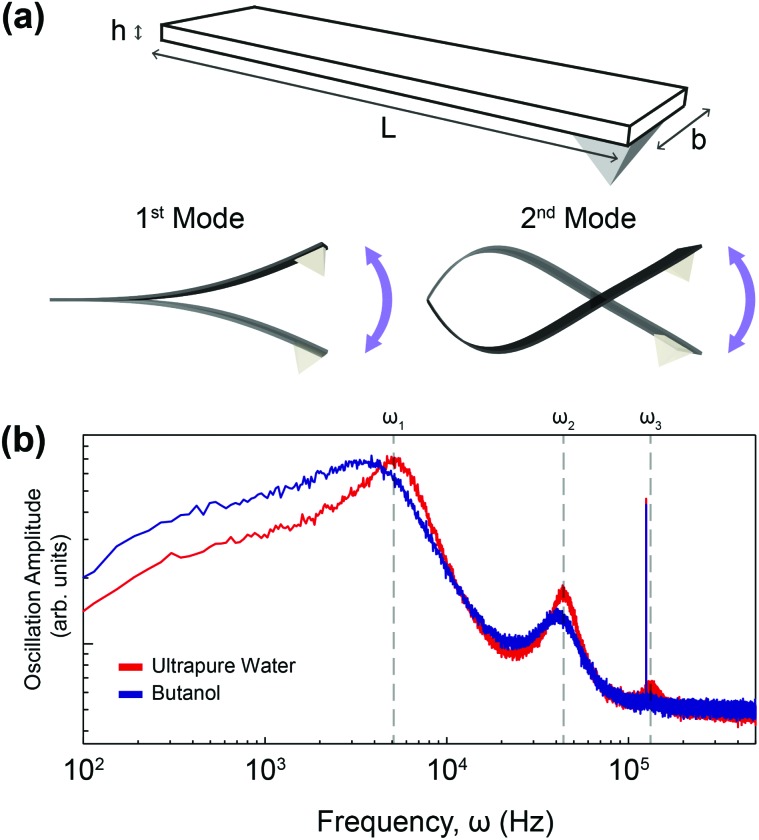
(a) Schematic of a cantilever with relevant dimensions (upper) and illustration of its first and second vibrational modes (lower left and lower right respectively). (b) Example of thermal spectra obtained from a cantilever immersed in ultrapure water (red) and in butanol (blue). Both spectra are displayed on the same log–log plot. The cantilever's first three eigenmodes (*ω*_1_, *ω*_2_, *ω*_3_) can be identified in water. In butanol, a shifting of the resonance frequencies to lower frequencies, together with the broadening of the resonance peaks is visible. The third eigenmode, *ω*_3_, can no longer be identified in the baseline noise, highlighting the importance of our model only requiring the first two modes.

The immersed microcantilever resonator is assumed to follow the Euler–Bernoulli formalism:1
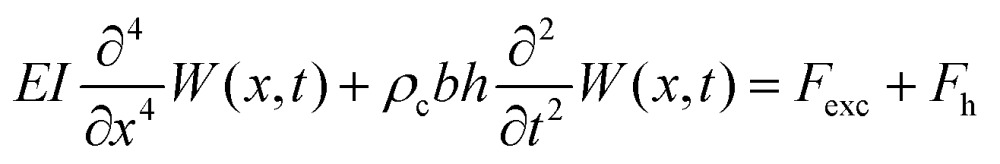
where *E* is the cantilever's Young-modulus, *I* is the rotary inertia of cantilever, *ρ*_c_ is the cantilever density, *L*, *b* and *h* are the length, width and thickness of the cantilever, respectively, *W*(*x*,*t*) is the time-dependent displacement of the cantilever, *F*_exc_ is the excitation force and *F*_h_ is the hydrodynamic force which can be described by a separate added mass and damping. Considering the added mass and damping per length of the cantilever^42,53,54^ and assuming a hydrodynamic function characterized by two real (*a*_1_, *a*_2_) and two imaginary (*b*_1_, *b*_2_) regression coefficients,^34^ we can relate the resonance frequencies of the cantilever in air, *ω*_an_, and in liquid, *ω*_fn_,^35,42,54^ for any given mode *n* (see the ESI† for details):2
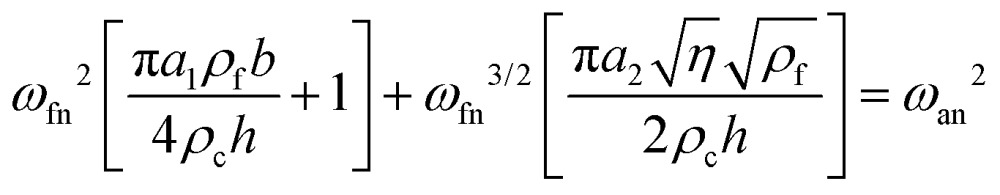
where *ρ*_f_ and *η* are the density and the viscosity of the fluid respectively. Using eqn (2), the coefficients of the real part of the hydrodynamic function can be calculated if the viscosity and density are known for a reference liquid (usually water). It requires measuring two resonance frequencies of the cantilever in air and in the liquid of interest (see the ESI†). Experimentally, the measurement of the lower resonance frequencies of the thermal spectra is easier, especially for stiffer cantilevers whose resonance frequencies can be relatively high. We therefore propose using the first two resonance frequencies, obtained directly from the cantilever's thermal spectrum (Fig. 1).

Using the resonance frequencies obtained experimentally for the reference liquid, here water, analytical expressions for the viscosity and density of an unknown fluid can then be derived:3
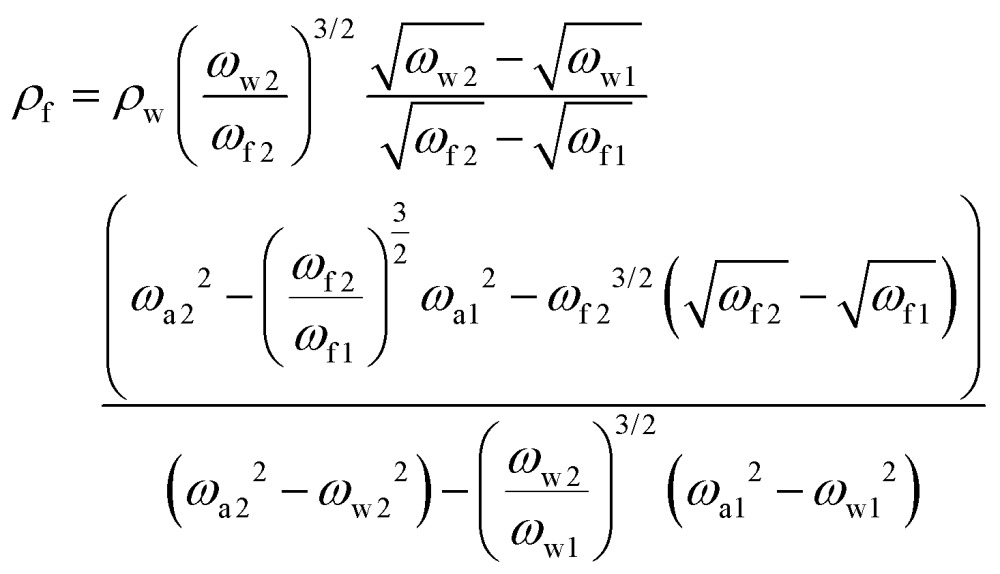

4
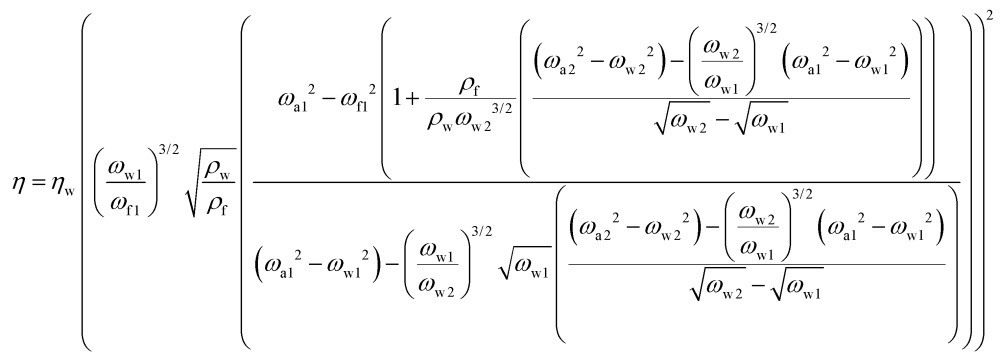
where the indices w, a, 1 and 2 correspond to water, air, the first resonance frequency and the second resonance frequency, respectively.

We note that any direct dependence on the geometrical parameters of the cantilever is cancelled out; the dependence is implicit in the hydrodynamic regression coefficients. Expressions (3) and (4) provide the core results for this paper but the derivation also provides analytical expressions for the quality factor (*Q*) of the different modes, as well as the added mass and damping to the cantilever at each frequency. Unlike the density and viscosity of the liquid, these quantities are expected to depend on the properties of the cantilever used for the measurement and hence provide a good opportunity to test the quality of the model.

## Experiments

**Fig. 2:**
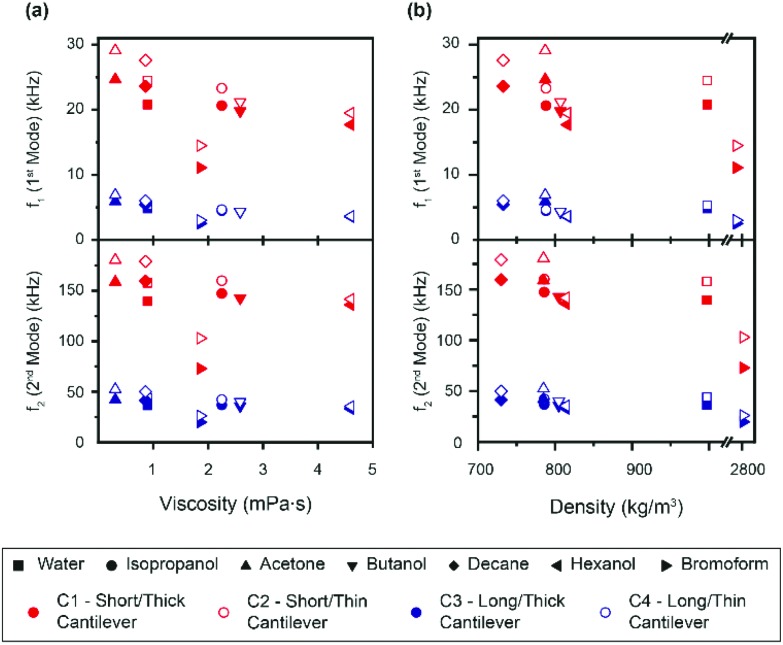
Measured resonance frequencies of the four different cantilevers in liquids of varying density and viscosity. The first and second resonance frequencies are plotted against the liquids’ viscosities (a) and densities (b). Overall, no obvious correlation is visible, except for a decrease in resonance frequencies with increasing density and viscosity. This decrease is however non-monotonic and its extent depends on the geometry of the cantilever used for the measurement.

In order to calculate the density and viscosity of a fluid from eqn (3) and (4), the two first resonance frequencies of the cantilever while immersed in the unknown fluid had to be measured (Fig. 1(b)). We therefore recorded the thermal spectra of the cantilever in the six liquids of interest, and in ultrapure water for reference. For each liquid, the measurement was repeated with four different cantilevers that exhibited different lengths, widths, and stiffnesses. The cantilevers are hereafter referred to as C1–C4 and their respective characteristics are presented in Table 1 (Materials and methods). This allowed us to examine the impact of the cantilevers’ properties on the calculated *ρ* and *η*. Fig. 2 shows the resonance frequencies of each cantilever in different fluids, plotted against the accepted^25,54,59,60^ viscosity (a) and density (b) of each fluid. From Fig. 2, it is clear that there is no obvious relationship between the resonance frequencies and either the viscosity or density of the surrounding liquid. Although increasing density and viscosity tends to reduce the resonance frequencies, the relationship is non-monotonic, and often different for the two resonances measured. The influence of the cantilever geometry is also evident, with shorter lengths exhibiting higher resonance frequencies. The effect of the cantilever's width is however less pronounced.

The accepted density and viscosity values for water at 25 °C are *ρ*_w_ = 997 kg m^–3^ and *η*_w_ = 8.94 × 10^–4^ kg m^–1^ s^–1^.^59^ For each cantilever, it is possible to calculate the hydrodynamic coefficients in water and use the accepted value as a calibration (see the ESI†). This can in turn be used to evaluate the added mass and added damping for the cantilevers in the different liquids investigated (ESI Fig. S1 and S2† respectively).

**Fig. 3:**
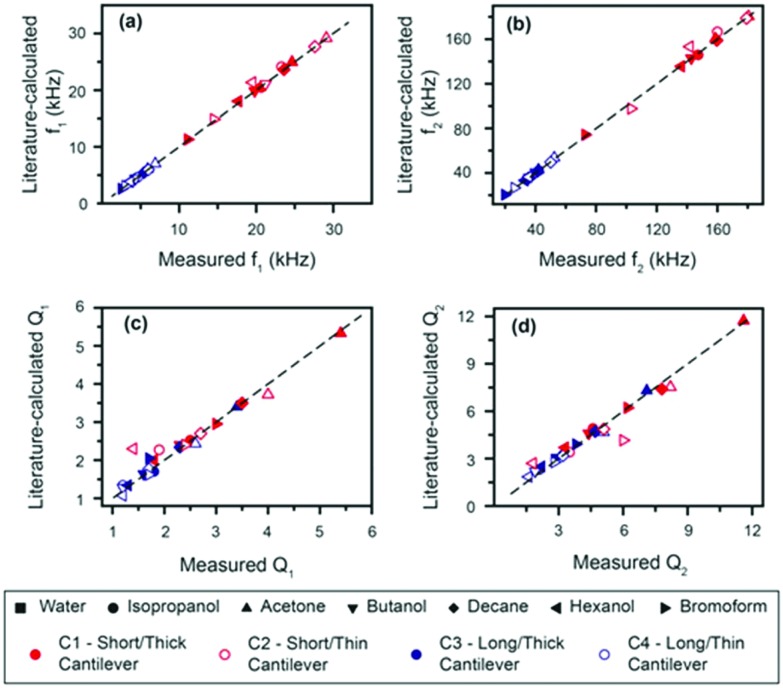
Comparison between the literature-calculated and measured frequencies ((a), (b)) and quality factors ((c), (d)) for different cantilevers in the test liquids. Lines with a gradient of unity are given as a guide to the eye to facilitate the evaluation. All points measured deviated from the calculated values by less than 3%, apart from hexanol, which has an error of less than 10%.

The accuracy of the derived expressions with water as a reference can be directly evaluated by comparing the measured resonance frequencies and quality factors of the cantilevers with those calculated (eqn (2) and S10†) using the accepted density and viscosity values (Fig. 3). We hereafter refer to the values calculated from accepted densities and viscosities as “literature-calculated”. Overall, the results show good agreement between the measured and literature-calculated values; a difference in frequencies of less than 3% except for hexanol, which shows the largest deviation (still <10%). Similarly, quality factors all show deviations smaller than 10% between the derived and measured values.

**Fig. 4:**
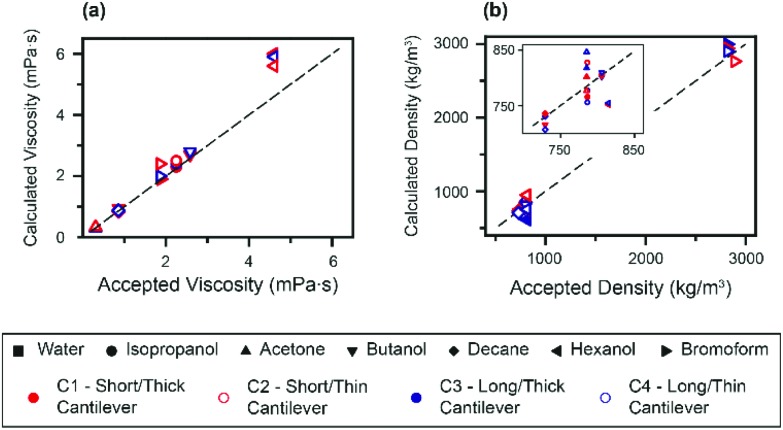
Comparison between the accepted and calculated viscosities (a) and densities (b) of the probed fluids. The values of *η* and *ρ* derived from the measurements compare well with the accepted values, as evidenced by their collapse onto the line of unity gradient. The inset in (b) highlights the data points at lower densities.

Finally, the expressions given by eqn (3) and (4) are used to derive the viscosities and densities of the different test liquids. The values obtained are directly compared with the accepted values for each liquid at 25 °C (ref. 25, 54, 59 and 60) in Fig. 4. As for Fig. 3, the error in the derived values is always smaller than 10%, which represents a significant improvement over previous approaches. Larger errors are incurred for liquids with higher viscosity, as illustrated by the data point for hexanol. This is somewhat to be expected, since the approximation for the hydrodynamic function^34^ is optimized for lower viscosities.

In practice, it is possible to improve the accuracy of the calculated viscosity and density by including the third resonance frequency in the hydrodynamic function. The density and viscosity of hexanol calculated from the second and third cantilever eigenmodes are shown in Table 2. Comparison with derivations using respectively the 1^st^/2^nd^ (*ρ*_12_, *η*_12_) and 2^nd^/3^rd^ (*ρ*_23_, *η*_23_) resonance frequencies shows that the latter works better for more viscous fluids.

**Table 2:** Percentage errors between the calculated and accepted values of density and viscosity for the more viscous liquids using the 1^st^/2^nd^ and 2^nd^/3^rd^ resonance frequencies. In all cases the long/thin cantilever was used. The error is reduced by considering the 2^nd^/3^rd^ resonance frequencies except for the density of butanol where the third resonance is difficult to identify (see Fig 1b)

	Error in *ρ*_12_ (%)	Error in *ρ*_23_ (%)	Error in *η*_12_ (%)	Error in *η*_23_ (%)
Isopropanol	3.8	1	10	8
Butanol	0.62	5	8.4	5.8
Hexanol	7.3	4.5	28.2	5

The results shown in Table 2 indicate that using the frequencies from the second and third eigenmodes of the cantilever tends to reduce the error. This is due to the fact that higher resonance frequencies are less sensitive to thermal noise when compared to lower modes. A thorough error analysis (ESI Fig S4†) validates this observation. However, using the third eigenmode requires the ability to measure it. Practically, this can be challenging in high-density fluids, especially for soft cantilevers. The current expression therefore provides a good compromise between accuracy, simplicity and practicality in most technological applications, and can readily be adapted to more viscous liquids if needed.

There is, however, an important point that has not been considered so far. Our model assumes that the viscosity is a scalar quantity and not a function of the probing frequency. In other words, we make the implicit assumption that the liquids probed are Newtonian. This assumption is mostly justified for the test liquids used to validate our model, but this may not hold, for example, for bodily fluids^4–6^ or lubricants.^1^ A deviation from Newtonian behaviour will induce some error in our predictions since the liquid is probed simultaneously at different frequencies, with the second frequency typically 5–6 times higher than the first. This could partially explain the poorer results obtained in the more viscous hexanol. In order to tackle this issue up front, we tested the model in ultrapure water solutions containing increasing concentrations of poly(ethylene) oxide (PEO), a simple uncross-linked polymer that has been shown to exhibit non-Newtonian properties in aqueous solutions.^37,39^ Specifically, these solutions are shear thinning across a broad range of molecular weights and concentrations,^40^ similarly to most bodily fluids. Practically, this means the cantilevers of different lengths will experience different rheological environments, with the shorter cantilever effectively subjected to a lower viscosity than its longer counterpart, as it oscillates at higher frequencies.

**Fig. 5:**
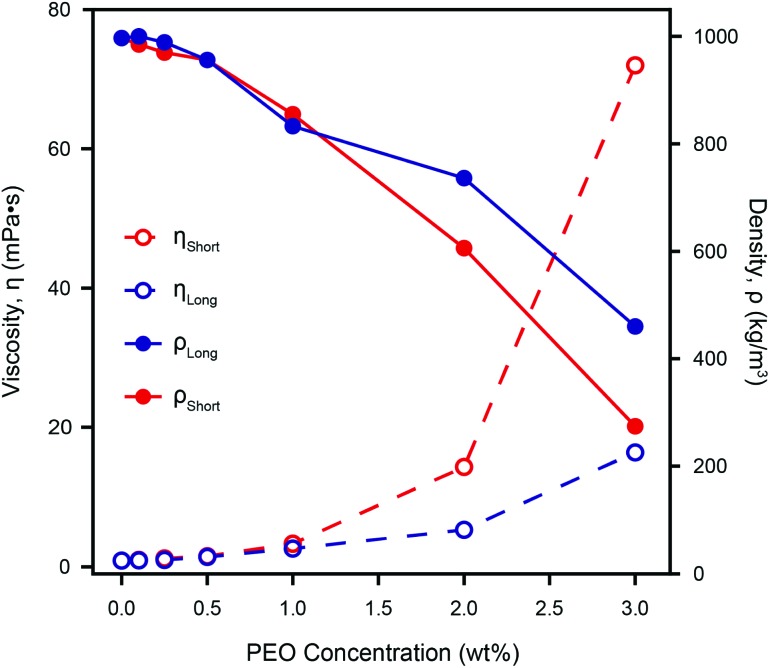
The calculated density, *ρ*, and viscosity, *η*, of different concentrations of PEO in ultrapure water as measured by two different cantilevers. Both *η*_Short_ and *η*_Long_ increase with increasing PEO concentration, but the effective viscosity measured by the shorter lever is always higher. The discrepancy between the two cantilevers increases with the PEO concentration due to a frequency dependence of the actual viscosity that is not accounted for by our model. However, *η*_Short_ agrees with standard rheometer measurements even for the highest PEO concentration measured.^61^ This demonstrates the validity of microcantilever measurements for non-Newtonian liquids, provided there is a suitable choice of a cantilever. The calculated density decreases as the concentration of PEO increases, and the discrepancy between the two cantilevers, although non-monotonic, increases in a similar manner to the viscosity. Comparison with the directly measured densities of the same weight-percent of PEO (see Fig. S5†) does not show a similar dramatic reduction of both *ρ*_Short_ and *ρ*_Long_, implying that the model's calculated densities are less robust than its viscosities.

Fig. 5 shows the density and viscosity of various dilutions of PEO in ultrapure water, as calculated using our model, with two cantilevers of different lengths (C1 and C3; subscripts “short” and “long” respectively). As the concentration of PEO increases, the viscosity and density derived from both cantilevers behave similarly; increasing and decreasing, respectively.

For relatively low PEO concentrations (<1.0 wt%), *ρ* and *η* as measured by each cantilever are similar, but at greater concentrations, the discrepancy increases dramatically. This indicates a strong dependence of the calculated values on the cantilever geometry and therefore the resonance frequency, as expected for non-Newtonian fluids. The fact that the observed discrepancy increases with PEO concentration is to be expected given that cantilevers are of different lengths (see Table 1) and therefore resonate at quite different frequencies – for example in ultrapure water, the resonance frequency of the first mode of the short cantilever is more than four times that of the long one. Our model depends on the ratio between the cantilever's eigenmodes and hence is particular sensitive to viscosity and density variations in non-Newtonian liquids.

For the shorter cantilever, the derived viscosity agrees very well with the standard rheometer measurements of PEO in fluid at concentrations of 2 and 3 wt%^61^ while for the longer cantilever the calculated viscosity values are significantly lower. The viscosity as measured by the shorter cantilever is always greater than that obtained from the longer cantilever, for solutions containing PEO. This reflects the fact that for longer cantilevers, the second and third modes of vibration were used as part of our model, due to the first mode being not measureable at high PEO concentration. This is in line with PEO's shear-thinning behaviour^40^ since the frequencies used for the longer cantilever are in fact higher than those used for the short cantilever. We therefore expect to find *η*_long_ < *η*_short_ as observed.

The measured density decreases monotonically with PEO concentration for both cantilevers, but the relationship between the two measurements is less straightforward than for viscosity. At PEO concentrations of nearly 0.5 wt% and 1.1 wt% the density measured by both cantilevers coincides. These concentrations are greater than the so-called overlap concentration *i.e.* the concentration above which the polymer coils are dense enough to form transient meshes.^37^ This suggests that the agreement may be due to nonlinearities in our model or possibly errors in determining the resonant frequency (see Fig. S4†), rather than reflecting the intrinsic properties of the polymer solution. At higher concentrations, the apparent reduction in density by a factor of over 3.5 (*ρ*_Short_) or over 2 (*ρ*_Long_) cannot be correct given the inclusion of only a few weight percent of the polymer. Indeed, independent density measurements, found no such dramatic change (see Fig. S5†). This suggests our model's calculated viscosity to be more reliable than the derived density, the latter becoming unphysical when probing non-Newtonian fluids.

Overall, we find that the discrepancy in our measurements in non-Newtonian PEO is comparable to that of previous methods in pure liquids. Furthermore, the use of several cantilevers provides an effective method for quantifying any deviation from the liquid's Newtonian behaviour, opening new possibilities of diagnostic devices.

## Conclusions

In this paper, we present a method to quantitatively determine the viscosity and density of different liquids from the thermal vibration of an immersed microcantilever. We derive analytical expressions based on the Euler–Bernoulli beam theory to quantify the surface-coupled hydrodynamic effect and deduce the viscosity and density of unknown fluids. Our method only requires measurement of the first two resonance frequencies of the immersed cantilever after calibration in air and in a reference liquid, here water. Significantly, the method implicitly takes into account the cantilever geometry in the calibration process, hence providing analytical expressions for the viscosity and density that are completely independent of the cantilever's characteristics. Experimental validation of the method over an extensive range of liquids yields errors of less than 10% with accepted values. The validity of the model in fluids with frequency-dependent viscosities, *η*(*ω*), was also investigated using PEO in different concentrations as a model non-Newtonian shear-thinning fluid. As expected, the method becomes progressively dependent on the cantilever geometry as the concentration of PEO increases. This is due to the fluid's viscosity becoming more dependent on the frequency as the density of the polymer chains increases. However, this dependence on the cantilever properties can be exploited to quantify the relative error of the measurement. Here, this error was in most cases less than 10% even for the liquid with behaviour comparable to bodily fluids.

We expect our results to contribute primarily to the development of lab-on chip devices and in nanofluidics. The method could also be used in the field of AFM in liquid, in particular in the analysis of surface-coupled effects on the cantilever vibrations and for the investigation of liquid flow near liquid–solid interfaces.
