# Supplementary material for: Simultaneous viscosity and density measurement of small volumes of liquids using a vibrating microcantilever
Source: Analyst. 2017 Mar 20;142(9):1492–8. doi: 10.1039/c6an02674e (PMC5450008; doi:10.1039/c6an02674e)
Supplement: Supplementary file 1 [file AN-142-C6AN02674E-s001.pdf]

Electronic Supplementary Material (ESI) for Analyst.
This journal is © The Royal Society of Chemistry 2017

Supplementary Information to

Simultaneous viscosity and density measurement of small volumes of liquids using a vibrating microcantilever

*Amir Farokh Payam, William Trewby, Kislon Voïtchovsky**

Department of Physics, Durham University, Durham, UK.

*electronic correspondence: kislon.voitchovsky@durham.ac.uk

Detailed derivation of the expression for viscosity and density

The microcantilever is modelled as a rectangular Euler-Bernoulli beam immersed in fluid environment (Fig 1). The following equation represents the microcantilever resonator immersed in liquid environment:

$$EI\frac{\partial^4}{\partial x^4}W(x,t) + \rho_c bh\frac{\partial^2}{\partial t^2}W(x,t) = F_{exc} + F_h \quad \text{(S1)}$$

where E is the cantilever's Young-modulus, I is the rotary inertia of cantilever, ρ_c is the cantilever density, L, b and h are length, width and thickness of the cantilever, respectively (fig. 1). $W(x,t)$ is the time-dependent displacement of cantilever, F_{exc} is the excitation force and F_h is the hydrodynamic force which can be described by a separate added mass and damping. The equations for added mass and damping per length of the cantilever are given by[1,2]:

$$m_h = \frac{\pi}{4}\rho_f b^2 \Re\{\Gamma(\omega_f)\} \quad \text{(S2)}$$

$$c_h = \frac{\pi}{4}\rho_f \omega_f b^2 \Im\{\Gamma(\omega_f)\} \quad \text{(S3)}$$

Where ω_f and ρ_f are the resonance frequency of the cantilever in liquid and the density of fluid, respectively. The hydrodynamic function is given as:

$$\Gamma = \Gamma_r + j\Gamma_i \quad \text{(S4)}$$

$$\Gamma_r = a_1 + \frac{a_2}{\sqrt{Re}}, \Gamma_i = \frac{b_1}{\sqrt{Re}} + \frac{b_2}{Re} \quad \text{(S5)}$$

Which a_i and b_i are regression coefficients of the hydrodynamic function[3,4].

The Reynolds number Re is given by:

$$Re = \frac{\rho_f \omega_f b^2}{4\eta} \quad \text{(S6)}$$

Where η is the viscosity of fluid.

The following equation can be used to obtain the resonance frequencies of the microcantilever in liquid[5]:

$$\omega_{fn}^2 \left[\frac{\pi a_1 \rho_f b}{4\rho_c h} + 1\right] + \omega_{fn}^{3/2} \left[\frac{\pi a_2 \sqrt{\eta}\sqrt{\rho_f}}{2\rho_c h}\right] = \omega_{an}^2 \quad \text{(S7)}$$

Where n is the mode number and κ_n is the wave number of cantilever. From equation (S7), given the values of viscosity and density for a reference liquid (usually water) and measurement of only two resonance frequencies of a cantilever in air and measurement fluid, the coefficients of real part of hydrodynamic function can be calculated. Since the proposed equation for hydrodynamic function is more accurate for the lower resonance frequencies[3], we propose to use the first two resonance frequencies of the cantilever for reconstruction of the hydrodynamic coefficients. The coefficients for real part of hydrodynamic function is obtained as:

$$a_1 = \frac{\left(\omega_{a2}^2 - \left(\frac{\omega_{f2}}{\omega_{f1}}\right)^{3/2} \omega_{a1}^2\right) - \omega_{f2}^{3/2}\left(\sqrt{\omega_{f2}} - \sqrt{\omega_{f1}}\right)}{\omega_{f2}^{3/2}\left(\frac{\pi \rho_f b}{4\rho_c h}\right)\left(\sqrt{\omega_{f2}} - \sqrt{\omega_{f1}}\right)} \quad \text{(S8)}$$

$$a_2 = \frac{\left(\omega_{a1}^2 - \omega_{f1}^2\right) - \left(\frac{\omega_{f1}}{\omega_{f2}}\right)^{3/2} \sqrt{\omega_{f1}} \left(\frac{\left(\omega_{a2}^2 - \omega_{f2}^2\right) - \left(\frac{\omega_{f2}}{\omega_{f1}}\right)^{3/2}\left(\omega_{a1}^2 - \omega_{f1}^2\right)}{\left(\sqrt{\omega_{f2}} - \sqrt{\omega_{f1}}\right)}\right)}{\omega_{f1}^{3/2}\left(\frac{\pi \sqrt{\eta}\sqrt{\rho_f}}{2\rho_c h}\right)} \quad \text{(S9)}$$

Equations (S8) and (S9) can be used not only for the case that cantilever vibrate far from the sample but also it can be applicable for the surface-coupled condition, which it needs only measuring first two resonance frequencies of cantilever in liquid without any data fitting.

The quality factors of cantilever in liquid is obtained as[5]:

$$Q_n = \frac{\omega_{f,n}(\rho_c b h + m_{hn})}{c_{hn}} \quad \text{(S10)}$$

By replacing equations (S2)-(S6), (S8) and (S9) in (S10) for first two eigenmodes of cantilever, the coefficients for imaginary part of hydrodynamic function given in equation (S5) are derived as:

$$b_1 = \frac{Re_2 \frac{\omega_{f2}}{Q_2}(\rho_c bh + m_{h2}) - Re_1 \frac{\omega_{f1}}{Q_1}(\rho_c bh + m_{h1})}{\sqrt{Re_2} - \sqrt{Re_1}} \quad \text{(S11)}$$

$$b_2 = \frac{\sqrt{Re_1 Re_2}\left(\sqrt{Re_1}\frac{\omega_{f1}}{Q_1}(\rho_c bh + m_{h1}) - \sqrt{Re_2}\frac{\omega_{f2}}{Q_2}(\rho_c bh + m_{h2})\right)}{\sqrt{Re_2} - \sqrt{Re_1}} \quad \text{(S12)}$$

After calculating these coefficients for a cantilever in a reference fluid, from equation (S7) and by only measurement of first two resonance frequency of cantilever immersed in fluid, the viscosity and density of fluid can be calculated with the following analytical equations:

$$\rho_f = \frac{4\rho_c h\left(\omega_{a2}^2 - \left(\frac{\omega_{f2}}{\omega_{f1}}\right)^{\frac{3}{2}}\omega_{a1}^2 - \omega_{f2}^{3/2}\left(\sqrt{\omega_{f2}} - \sqrt{\omega_{f1}}\right)\right)}{\pi a_1 b \omega_{f2}^{3/2}\left(\sqrt{\omega_{f2}} - \sqrt{\omega_{f1}}\right)} \quad \text{(S13)}$$

$$\eta = \left(\frac{2\rho_c h\left(\omega_{a1}^2 - \omega_{f1}^2\left(1 + \pi\rho_f b a_1/(4\rho_c h)\right)\right)}{\omega_{f1}^{3/2}\pi a_2\sqrt{\rho_f}}\right)^2 \quad \text{(S14)}$$

To calculate viscosity and density from (S13) and (S14), the dependency on geometrical parameters of cantilever is cancelled out. For example, in the case that the water is considered as reference liquid, the density and viscosity of other fluids can be calculated from following equations:

$$\rho_f = \rho_w\left(\frac{\omega_{w2}}{\omega_{f2}}\right)^{3/2}\frac{\sqrt{\omega_{w2}} - \sqrt{\omega_{w1}}}{\sqrt{\omega_{f2}} - \sqrt{\omega_{f1}}}\frac{\left(\omega_{a2}^2 - \left(\frac{\omega_{f2}}{\omega_{f1}}\right)^{\frac{3}{2}}\omega_{a1}^2 - \omega_{f2}^{3/2}\left(\sqrt{\omega_{f2}} - \sqrt{\omega_{f1}}\right)\right)}{(\omega_{a2}^2 - \omega_{w2}^2) - \left(\frac{\omega_{w2}}{\omega_{w1}}\right)^{3/2}(\omega_{a1}^2 - \omega_{w1}^2)} \quad \text{(S15)}$$

$$\eta = \eta_w\left(\left(\frac{\omega_{w1}}{\omega_{f1}}\right)^{3/2}\sqrt{\frac{\rho_w}{\rho_f}}\left(\frac{\omega_{a1}^2 - \omega_{f1}^2\left(1 + \frac{\rho_f}{\rho_w\omega_{w2}^{3/2}}\left(\frac{(\omega_{a2}^2 - \omega_{w2}^2) - \left(\frac{\omega_{w2}}{\omega_{w1}}\right)^{3/2}(\omega_{a1}^2 - \omega_{w1}^2)}{\sqrt{\omega_{w2}} - \sqrt{\omega_{w1}}}\right)\right)}{(\omega_{a1}^2 - \omega_{w1}^2) - \left(\frac{\omega_{w1}}{\omega_{w2}}\right)^{3/2}\sqrt{\omega_{w1}}\left(\frac{(\omega_{a2}^2 - \omega_{w2}^2) - \left(\frac{\omega_{w2}}{\omega_{w1}}\right)^{3/2}(\omega_{a1}^2 - \omega_{w1}^2)}{\sqrt{\omega_{w2}} - \sqrt{\omega_{w1}}}\right)}\right)\right)^2 \quad \text{(S16)}$$

where the indices w, a, 1 and 2 stand for water, air, first resonance frequency and second resonance frequency, respectively. The presented equations are completely independent of cantilever parameters, which increases the accuracy of calculation and removes the dependency related to cantilever geometry.

Additional Results and Discussion

When the cantilever vibrates in liquid environment, due to the viscosity and density of fluid, the cantilever behavior is affected by surrounding liquid which are described by added mass and damping. To investigate the effect of viscosity, density and cantilever properties on the added mass and damping of cantilever, the added mass and added damping for each cantilever in different liquid mediums are calculated from equations (S2)-(S3), multiplying by the length of the cantilever. The results are shown in figures S1 and S2 respectively.

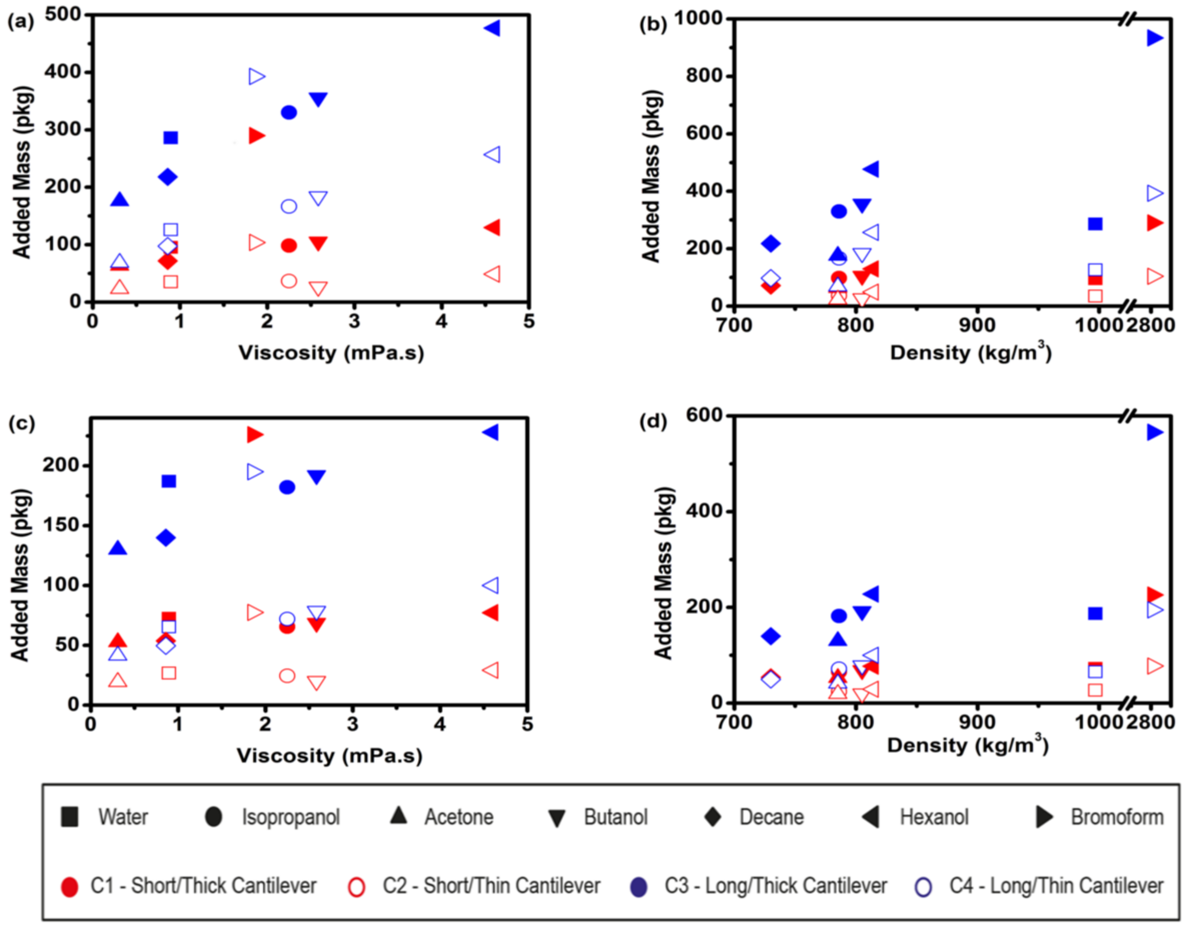

Fig. S1. Added mass for different cantilevers and liquids. The added mass for the first (respectively second) eigenmodes are plotted against viscosity (a, respectively c) and density (b, respectively d).

The results in Fig. S1 shows that the added mass varies non-monotonically with viscosity for both the first and second resonances. There is also a clear impact of the liquid's density on the added mass, but a better-defined trend appears in the dependence on viscosity. Generally, a higher resonance mode tends to present less added mass. This indicates that at higher frequencies, the effect of inertial load over the cantilever is reduced. This is in contrast to the effect of damping which increases with higher modes (Fig. S2). When considering the added

damping, the trend is also far clearer in viscosity than in density, suggesting the former to be the dominating factor.

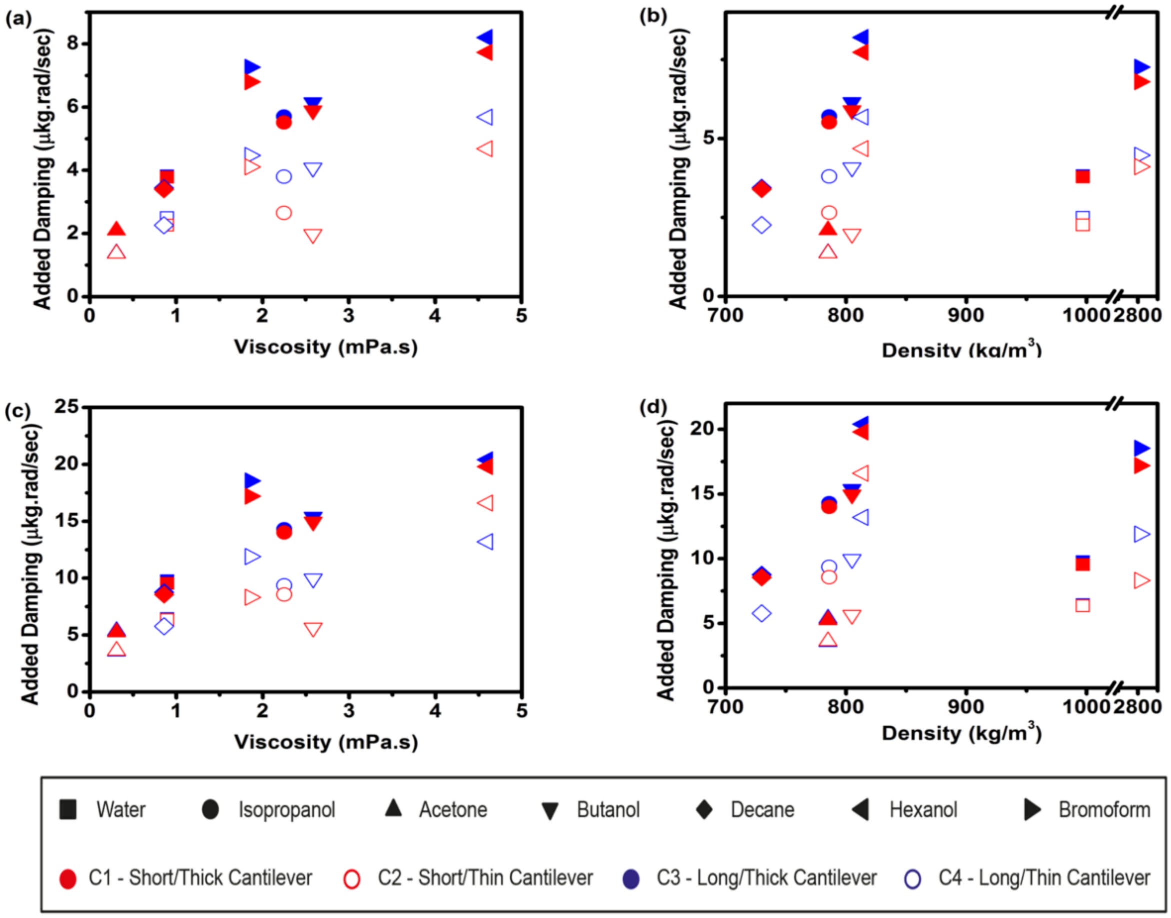

Fig. S2. Added damping for different cantilevers and liquids. The added damping for the first (respectively second) eigenmodes are plotted against viscosity (a, respectively c) and density (b, respectively d)..

The effect of cantilever geometry on the hydrodynamics is also visible in figures S1 and S2. The results indicate that the longer and wider a cantilever is, the greater the added mass. For the higher resonances and in the liquids with lower viscosity, the influence of length is more significant than that of the width (Fig. S1). This is no longer true when considering the added damping for which the cantilever width is more important.

The effect of added mass and damping on the frequency response of the cantilever is given in figure S3. It is immediately visible that the resonance frequency decreases with added mass, regardless of the damping. In other words, the contribution of added mass dominates any shift in frequency of microcantilever when compared to added damping. The effect of added damping is however more pronounced for higher resonances than for lower modes.

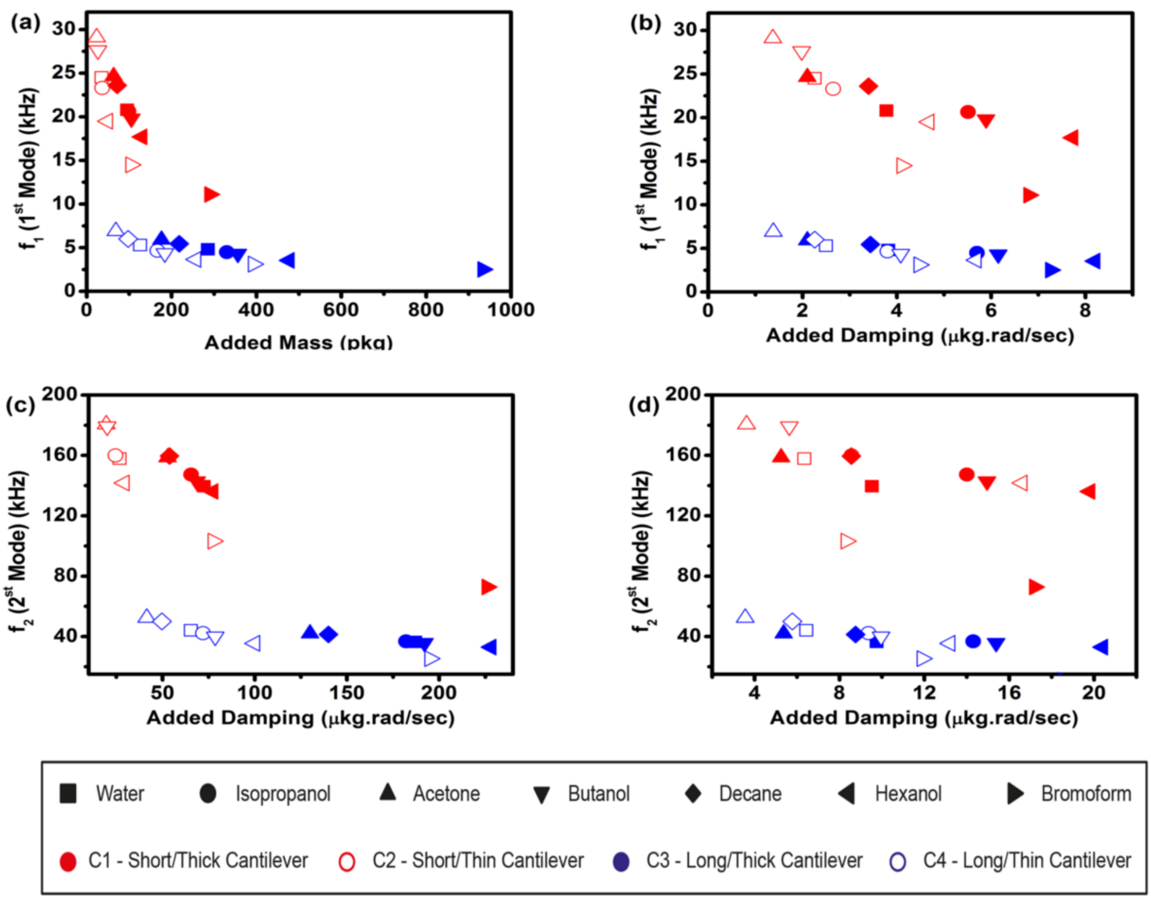

Fig. S3. Resonance frequencies of microcantilever versus added mass and added damping for the first mode (a) and (b), and the second mode (c) and (d).

Robustness Analysis

To investigate, the effect of frequency variation on the calculation of density and viscosity, we have performed error analysis. To do this, we calculate the range of frequency variations of the first and second modes for which the discrepancy between the calculated and accepted values of viscosity and density remains within 10%. The results presented in Fig. S4 show that the second mode's resonance frequencies can vary to a much greater extent while still producing viscosities and densities accurate to within 10 %. This implies the method is more sensitive to the frequency change in the first mode in comparison with second mode. Also, using short cantilever gives more robustness to the frequency variations in comparison with long cantilevers as it resonates at higher frequencies. Based on these results we can summarize that to improve the accuracy of calculation, it is recommended to use cantilevers with higher resonance frequencies.

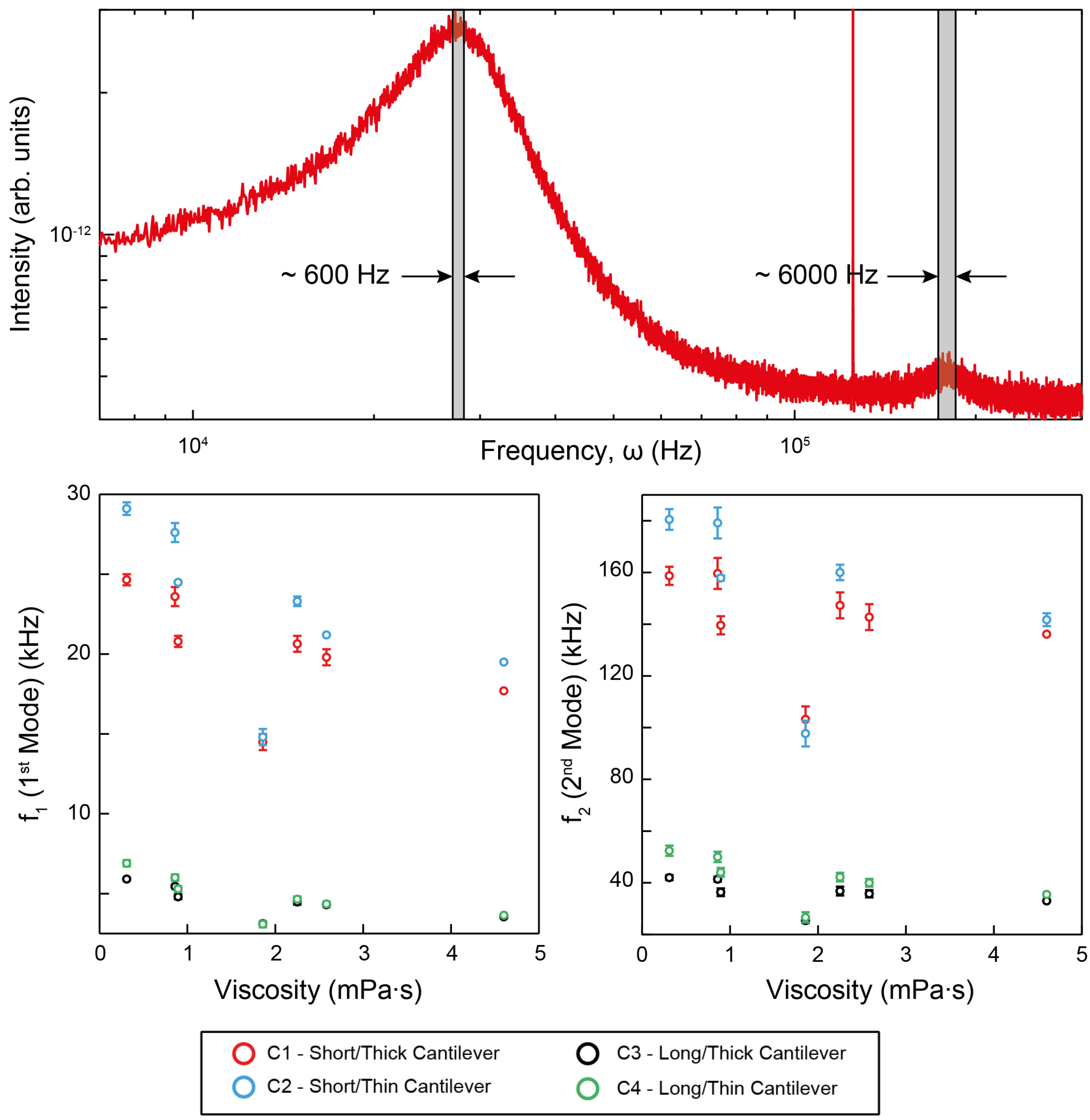

Fig. S4. Illustration of the how robust our model is to fluctuations in the first and second resonance frequency. (Upper panel) Example thermal spectrum (red) on a log-log plot with segments (grey) showing the range in which the resonance frequencies can vary while still producing viscosities and densities within 10% of their accepted value. (Lower panels) Recreation of figure 2(a) from the main text, but with bars representing the robustness measurements described above. Clearly the higher frequencies (short cantilever and/or 2nd resonance mode) are more robust with respect to frequency variations.

Independent PEO Density Measurements

In order to evaluate our model's calculated density for the various solutions of PEO, we prepared more aqueous solutions of the polymer as described in the main text. We then performed a direct measurement of the mass of different volumes (100 µL, 250 µL, 500 µL

and 1000 μL) of each concentration. The data were then fitted with a first-order polynomial (Igor Pro software v6.37, Wavemetrics) and the gradient and its uncertainty were extracted to find the density of the solutions. The results are displayed in figure S5. Despite the scatter of the data and the uncertainty on each point being very large, there is clearly no indication of a remarkable decrease in fluid density as predicted by our model (as in figure 5 of the main text). This implies that the density predicted by our model very quickly becomes inaccurate when used with viscoelastic polymer solutions.

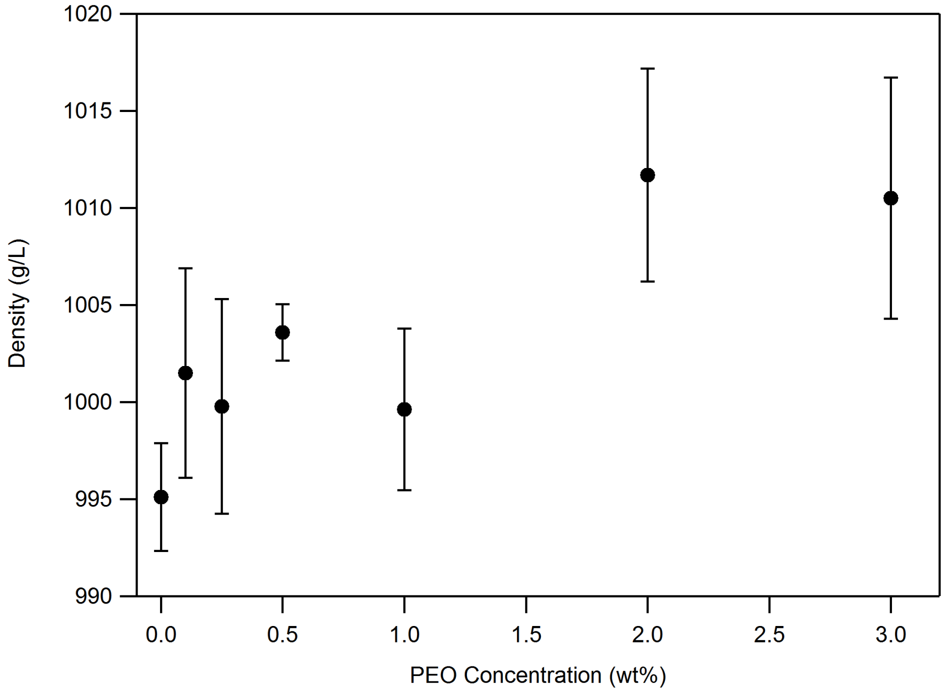

Figure S5: Independent, direct measurements of the density of varying concentrations of PEO. The error bars represent the uncertainty in the linear fit of the mass/volume data (see text). The data do not seem to have a clear trend, especially given the relatively large uncertainty on each point. However, the existence of a decrease in ρ by a factor of > 2 can be firmly ruled out, highlighting the large inaccuracies of our model at high weight percentages of PEO.

Supplementary References

1. A. F. Payam and M. Fathipour, *Micron* 2015, **70**, 50-54.
2. D. Kiracofe and A. Raman, *J. Appl. Phys.,* 2010, **107**, 033506.
3. A. Maali, C. Hurth, R. Boisgard, C. Jai, T. C. Bouhacina and J. P. Aime, *J. Appl. Phys.,* 2005, **97**, 074907.
4. R. C. Tung, J. P. Killgoreand D. C. Hurley, *J. Appl. Phys.,* 2014, **115**, 224904.
5. A. F. Payam, *Ultramicroscopy,* 2013, **135**, 84-88.
